# Learnt effects of environmental cues on transport-related walking; disrupting habits with health promotion?

**DOI:** 10.1371/journal.pone.0220308

**Published:** 2019-08-01

**Authors:** Frank F. Eves, Anna Puig-Ribera

**Affiliations:** 1 School of Sport, Exercise and Rehabilitation Sciences, University of Birmingham, Birmingham, United Kingdom; 2 Departament de Ciències de l´Activitat Física, Universitat de Vic-Universitat Central de Catalunya, Spain; The University of Tokyo, JAPAN

## Abstract

**Background:**

In Ecological models, physical environments can be important determinants of transport-related walking. With repeated exposure to the same environment, learning of a linkage between the cues in the environment and walking should occur. Subsequent encounters with the cues can prompt the behaviour relatively automatically. No studies have experimentally tested the potential learning of this linkage between cues and behaviour. Choices between stairs and escalators in public access settings were employed to test this premise for transport-related walking.

**Methods:**

Three studies investigated the effects of visual cues on stair/escalator choices (combined *n* = 115,062). In quasi-experimental, interrupted time-series designs, observers audited choices in public access settings. *Design alone* phases with art or coloured backgrounds were compared with *design plus message* phases in which verbal health promotion messages were superimposed on the visual cues. Analyses used bootstrapped logistic regression.

**Results:**

In initial studies, the *design alone* phases had no effect whereas subsequent *design plus message* phases reduced escalator choice. In two further studies, a 5–6 week *design plus message* phase that reduced escalator choice preceded a *design alone* phase. The visual background behind the successful health promotion message was reintroduced four weeks after the intervention was removed. The visual cue of *design alone* reduced escalator choice after it had been paired with the verbal health promotion message. There were no differences between art and coloured backgrounds.

**Conclusion:**

These studies demonstrate for the first time a learnt linkage between transport-related walking and environmental cues. Discussion focuses on the mechanisms that may underlie this learning and cues in the environment that are relevant to transport-related walking.

## Introduction

Unhealthy lifestyles increase the population burden of disease [1]. Smoking, diet, lack of exercise and alcohol consumption all contribute to ill-health. Critically, the ill-effects of lifestyle rarely result from a single episode of the unhealthy behaviour. Rather, repetition of the behaviour produces cumulative effects on health. In common parlance, these unhealthy behavioural choices are often described as *bad habits* to denote the fact that they appear as a regular occurrence within an individual’s life. Habits can be operationalised as impulses to action triggered by contextual cues that have been associated with behavioural choices that succeed in their goal [2–7]. This paper experimentally tests the effects of pairing contextual cues with a successful health behaviour to disrupt a less healthy habitual choice, namely choosing the escalator instead of the stairs.

Choice of stairs rather than escalators is a current health promotion target to increase physical activity as part of daily life [1]. Stair climbing is a vigorous lifestyle activity, requiring 9.6 times the energy expenditure of the resting state [8]. In the seminal Harvard Alumni studies, stair climbing was protective for coronary heart disease and stroke [9,10]. Subsequent experimental studies reported that increased stair climbing improved cardiorespiratory fitness, lipoproteins, blood pressure and body fat, major risk factors for cardiovascular disease [11–15]. Even brief bouts of climbing increase cardiorespiratory load. Based on heart rate during climbing, the vigorous intensity threshold is reached after 21s of climbing [11,12], the time taken to ascend around 6m on the stairs [16]. Stair climbing is a time-efficient means of reducing cardiovascular disease risk that can be accumulated as part of daily life [11]. In contrast, pedestrians who avoid stair climbing by choosing the escalator forgo an opportunity to reduce disease risk. In shopping malls, 92.4% choose the less healthy option and avoid climbing [17].

Simple interventions, called point-of-choice prompts, can reduce these less healthy choices by pedestrians. Health promotion signage, installed at the point of choice between the stairs and the escalator, consistently increases the proportion choosing the healthier option of the stairs [18–23]. These simple interventions interrupt habitual choice of the escalator [24–26]. Current health psychology is particularly interested in habits because of their potential importance to health behaviour and interventions (see [3,27]). This paper test one of the mechanisms said to underly habitual behaviour, namely its link to contextual cues. Research on the development of habits in humans contrasts thoughtful deliberation about behaviour when a new situation is encountered with more automatic processing with repetition of the same situation [4,5]. A successful previous choice in the same situation will encourage its repetition [5] and reinforcement of any choice by success inevitably reinforces the contextual cues associated with that option. Subsequent encounters with the cues may prompt the behaviour in a relatively automatic manner [3,7,28–34]. Nonetheless, this proposal for contextual cueing has been made without directly testing the predicate of the model that repetition of environmental cues can produce learnt behaviour. Only two recycling studies have explored experimentally the effects of a cue on behaviour. In one study, effects of the cue appeared almost instantaneously, an implausible timeframe for habit development [35] whereas in the other study, the cue itself had no effects on behaviour in modelling [36]. This paper provides experimental evidence for the learning of environmental cues that influence one component of transport-related walking, namely stair climbing. We disrupt the less healthy habit of escalator choice to test whether this disruption is linked to the cues that accompany the change to a healthier option.

The paper reports data from three studies of choice between the stairs and an escalator in public access sites. This naturally occurring encounter with contextual cues occurs when pedestrians transport themselves between contexts. The studies here used a visual cue, signage installed in the environment, to disrupt habitual choice in an invariant location. Choice between stairs and an escalator is biased. Humans naturally minimize the energetic cost of walking [17,37]. Structural aspects of any stair/escalator complex will influence the behaviour. The greater the height of the climb, the less likely it is that individuals will choose to climb [38,39]; a greater height of climb entails greater energy expenditure when raising body mass against gravity. In addition, stairs that represent a more direct route to the destination are more likely to be chosen [25,40]; the direct route to a destination reduces both the energetic and temporal costs of the journey. Typically, when confronted with a choice between stairs and an escalator, pedestrians reduce the energetic cost of the journey by choosing the escalator; in shopping malls 92.4% avoid the stairs [17]. The consistency of the context, and the reward of energy minimization, allows habitual avoidance of stairs by choosing the escalator to develop. This avoidance is a simple, naturally developed response to environmental cues that requires only the ‘minimal or sporadic monitoring’ characteristic of habitual behaviours (page 1287, [41]). If, however, verbal health promotion messages are installed as prompts at the choice point between stairs and escalators, habitual choice can be disrupted and some pedestrians choose the stairs [24,42]; 37/41 studies have been successful (see [16,20,21]). The contextual change produced by point-of-choice prompts promotes deliberative processing of any verbal health promotion message superimposed on the visual cue [24,25]. Those pedestrians with intentions to be more physically active can respond to the prompt in the environment to satisfy this goal [16,43,44]. The successful behaviour that was used to test the learning of the new contextual cues was this increased stair climbing in response to the health promotion message in some pedestrians.

### Overview of studies

All studies employed quasi-experimental, interrupted time series designs. An initial baseline phase was followed by subsequent intervention and sometimes new baseline phases. Uniformly, the interventions employed prompts that changed the environmental cues at the point-of-choice between the escalator and the stairs. The health promotion message ‘*Take the stairs*. *7 minutes of stair climbing a day protects your heart/health’* was used throughout to disrupt escalator choice. In all studies, two different intervention phases were employed; a phase without any verbal message, *design alone*, was contrasted with a phase where health promotion messages were superimposed on the design, *design plus message*. With one exception, the designs used pictorial elements, i.e. art, as the background. Visibility of the intervention is a key component of success with point-of-choice prompts [45–47]. We reasoned that large, art-based designs would be more likely to attract attention and hence disrupt habitual choice.

In the initial studies, art-based designs *without any message* were installed first at the point-of-choice. Changes to the appearance of workplace stairwells have been associated with increased stair use [48,49]. These initial studies tested whether changes to the environment with art-based designs alone would disrupt escalator choice in public access settings. The absence of any effects of *design alone* is a necessary pre-condition for any test of cue-learning. In the subsequent studies, the order of the phases was reversed. Thus, the *design plus message* phase was presented first to allow pairing of the visual cues with changes in behaviour promoted by the health message. Following this learning phase, the *design alone* was presented after a 4-week no-intervention period to test whether the cues alone would change behaviour. The overall aim of these studies was to test whether transport-related walking could be linked to learning of new environmental cues; this aim was achieved. Table 1 summarises the sequence of phases of the different studies presented in this paper.

**Table 1:** Summary of the sequence of different phases of the studies reported in the paper.

Site	Cue type	Cue area	Weeks																
			1	2	3	4	5	6	7	8	9	10	11	12	13	14	15	16	17
Monjuic outdoors Barcelona (2005)	Art	2.8m^2^	█	█	█	█	█	█											
Leiden station (2008–2009)	Art	2.5m^2^	█	█	█	█	█	█	█				█	█	█	█	█	█	
Clot station Barcelona (2010)	Art	2.0m^2^	█	█	█	█	█	█	█	█					█	█	█	█	█
Mundet station Barcelona (2010)	Colour	2.0m^2^	█	█	█	█	█	█	█	█					█	█	█	█	█

Baseline = █ Design alone = █ Design + message = █

### Methods common to all studies

Ethical approval for all the studies was obtained from the ethics subcommittees of the School of Sport and Exercise Sciences, University of Birmingham, University of Vic, Catalunya and TNO, Leiden. The ethics committees did not require the consent of observed pedestrians. We obtained all required permits and approvals to conduct research in the Netherlands. Throughout the study periods, inconspicuous observers coded pedestrian choices and the associated demographics at the sites using a standard protocol employed in previous research [24]. Typically, demographic composition and pedestrian traffic volume influence choice independently of any intervention (S1 File for further information about demographics and sample composition for each study). In all studies, children and individuals accompanied by children were excluded from analyses, consistent with previous research [24]. The reported Odds Ratios (OR) for the dichotomous outcome variable of escalator choice from multiple logistic regression represent the effects of the cues corrected for the uncontrollable influences of demographics and pedestrian traffic. Analyses employed bootstrapping with replacement (samples = 1000). This approach summarises the results of 1000 separate analyses with different samples from the data set to circumvent problems of potential non-independence of the observations.

## Effects of *design alone* before pairing with health promotion messages

## Study 1

## Methods

Study one was conducted at an outdoor site in the Monjuic region of Barcelona, Catalunya in Spain (October–November, 2005). At this site a 36-step staircase (height of climb = 5.94m) was separated from the up and down escalator by a 0.82m wide, grey concrete wall, with the up escalator adjacent to the wall. The stairs and escalator provided access to a raised walkway crossing a major road. Observations were made from 10.00am—1.00pm and from 3.00pm– 5.00pm, two days a week (*N* = 5,987). Due to an oversight, observations were not coded into separate 30 minute periods that would have allowed calculation of pedestrian traffic volume for each 30 minutes to include in analyses (*c*.*f*. [24]).

### Intervention

**Fig 1:**
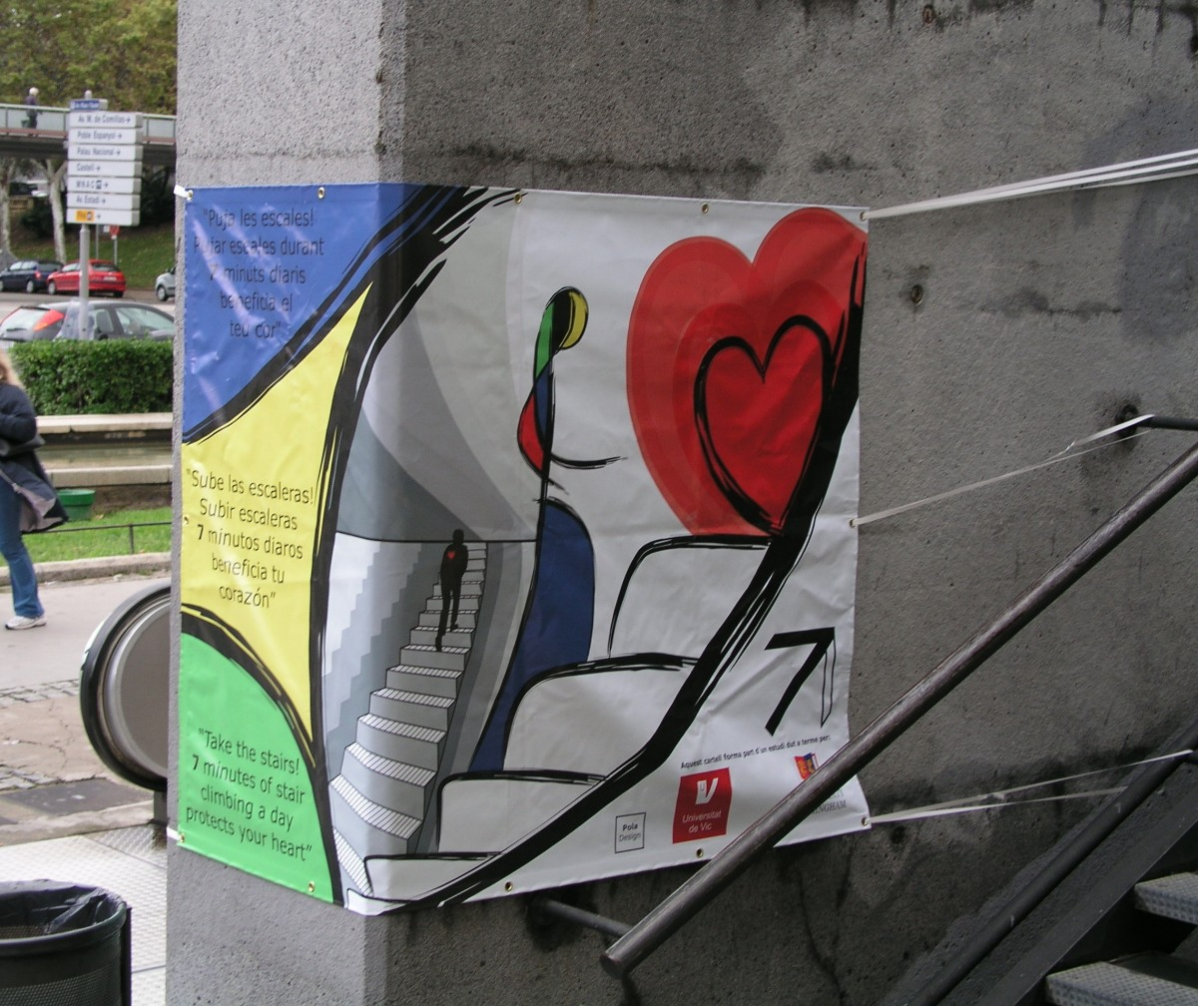
Intervention poster with message at the outdoor site in Monjuic, Barcelona.

A two-week baseline was followed by a two-week *design alone* phase. The design was a 1.4m high x 2m long waterproof banner affixed to the concrete wall at head height with a continuation of the design on the side of the wall with the stairs. The section of design facing pedestrians approaching the site contained blocks of colour and the start of a set of stairs. The continuation of the banner on the stair side of the wall depicted a stylised woman climbing stylised stairs towards a large red heart, with the design completed in the style of Miro by a Catalan artist (Josep Mª Rius Graells). This section of the design was hidden from approaching pedestrians. For the *design plus message* phase (two weeks), the message in English ‘*Take the stairs*! *7 minutes of stair climbing a day protects your heart*’ and translations of this message into Catalan ‘*Puja les escales*! *Pujar escales durant 7 minutos diaris beneficia el teu cor*’ and Spanish ‘*Sube las escaleras*! *Subir escaleras 7 minutos diaros beneficia tu corazón*’ were superimposed on the section of the banner facing the approaching pedestrians (see Fig 1). This message was based on Yu and co-workers estimate that the volume of vigorous intensity exercise required to reduce all-cause mortality by 50% in middle aged men was equivalent to seven minutes of stair climbing a day [50]. The message successfully increased stair climbing at public access sites in Barcelona [51] and the UK [25], as well as in a UK workplace [52].

## Results

Table 2 which summarises the percentage of escalator choices during the different phases reveals high rates of stair avoidance at the Monjuic outdoor site. Comparison of the baseline with the *design alone* phase revealed no effect of the design on escalator use (OR = 1.21, 95% Confidence Intervals (CIs) = 0.49, 2.99, *p* = .69). In contrast, addition of the health promotion message decreased escalator use relative to the previous phases (OR = 0.55, CIs = 0.31, 0.98, *p* = .04). There were no other statistically significant effects with this relatively small sample.

**Table 2:** Percentage escalator use (95% CI) in each phase for study 1 and equivalent data from a UK replication reported elsewhere [25].

Site	Location	Cue type	Cue area	Baseline	*Design alone*	*Design + Message*
Barcelona Monjuic (2005)	Outdoors	Art	2.8m^2^	99.3%^a^ (98.6, 99.7)	99.3% (98.8, 99.8)	98.9% (98.3, 99.3)
Coventry, UK (2004)	Shopping mall	Art	3.6m^2^	96.3% (95.9, 96.6)	96.8% (96.4, 97.1)	90.9% (90.2, 91.4)

^a^ The table contains raw percentages, uncorrected for effects of demographic grouping and pedestrian traffic volume.

## Discussion

In a planned replication with a considerably larger sample (n = 40,057) reported elsewhere [25], an art-based design in the style of Mondrian covered the whole of the stairs (see summary in S2 File). As in Monjuic, there were no effects of the *design alone* phase (see Table 2). Neither of these initial studies revealed any change in behaviour in the *design alone* phase yet escalator choice was reduced when a health promotion message was superimposed on the design. The discrepancy between these null effects on public access staircases and reported effects of modification to the stairwell in workplaces [48,49] may simply reflect the different settings [20,21]. For public access staircases, the cues would be visible to stair and escalator users alike as both methods of ascent were adjacent. In the workplace studies, the stairs were remote from the elevators and hence pedestrians had to enter the stairwell to view the changed environmental cues. One point is clear. Replicated data from two different countries provide no evidence that colourful designs alone that change visual cues within the environment disrupted choice of the escalator. Hence, testing the effects of pairing a verbal health promotion messages with visual cues was possible.

## Effects of *design alone* after pairing with health promotion messages

In the remaining studies, the order of the phases was reversed. Thus, a *design plus message* phase preceded a *design alone* phase installed four weeks after removal of the initial intervention. Additionally, a second baseline phase was inserted between the *design plus message* and *design alone* phases. A summary of the design elements has been presented earlier in Table 1. These studies tested whether pairing of the visual cue with a health promotion message would result in disruption of escalator choice when the *design alone* phase was reintroduced. We predicted that successful health promotion during the *design plus message* phase would be followed by disruption of escalator choice when the contextual cue of the *design alone* phase was re-introduced.

Before presenting these studies, two points should be made explicit. It is already clear that learning occurs with point-of-choice prompts; three months after removal, rates of escalator use can remain reduced relative to the original baseline periods [24,53]. Therefore, a second non-intervention measurement period was included immediately prior to installation of the *design alone*. Further, as is clear from the preceding studies, only some pedestrians respond to the original intervention. Those wishing to change their behaviour will be a restricted subset of the observed population; some intention to change is a pre-requisite for response to a point-of-choice prompt [16,43,44]. As a result, it seems unlikely that effects of the *design alone* phase could exceed the disruption produced by the original verbal message. Those pedestrians who originally respond seem the likely candidates for the learning of the cues as only they have performed the successful health behaviour in response to the *design plus message*. In this quasi-experimental design where health information must precede tests of the effects of *design alone*, removal of the message cannot remove the explicit knowledge about health that it provided. Rates of escalator use may not return to a level sufficiently above that of the original intervention phase and impede testing for effects of environmental cues alone.

Two preliminary studies, reported in supporting materials (S2 File), tested for potential effects of environmental cues after pairing with a health promotion message. In a UK shopping mall, a 13-week *design plus message* phase was followed by a one-week *design alone* phase beginning five weeks after the main intervention in which coloured backgrounds were affixed to the stairs. Introduction of the *design alone* had no effect on escalator use. For pedestrians in a shopping mall, it is unclear how many times they would use the site. As the number of repetitions required to link contextual cues to behaviour is unknown, it is possible that insufficient pairings of the visual cues with the message occurred, despite the 13-week intervention. For the next study in a Barcelona train station, morning commuters were presented with a 6-week *design plus message* phase and a *design alone* phase introduced, three weeks after removal. There were no statistical differences between the second baseline and the *design alone* phase. It was possible that disruption of escalator use by the health promotion message had not dissipated sufficiently to test effects of the cue alone. In addition, the layout of the site biased choice towards the escalator (see S2 File).

The next station study employed a 4-week gap between *design plus message* and *design alone* phases. In this study, monitoring performed both before and after the *design alone* phase was able to test the specificity of effects during *design alone*.

## Study 2

## Methods

The site was an overland station in Leiden, the Netherlands in which the monitored platforms served trains to Amsterdam and Den Haag, i.e. commuter trains (November 2008—February 2009). Pedestrians ascending to the platforms choose either an adjacent escalator or 36-step staircase (height of climb = 6.12m). The stairs/escalator could be approached from either the left (stairs reached first) or the right (escalator reached first). A single observer recorded choices between 7.00 and 8.50 am, two days a week (*N* = 37,479), as well as their direction of approach to reach the stair/escalator complex (left vs. right). Pedestrian traffic volume was operationalised as the total number of passengers arriving for each train using both the stairs and escalator.

### Intervention

**Fig 2:**
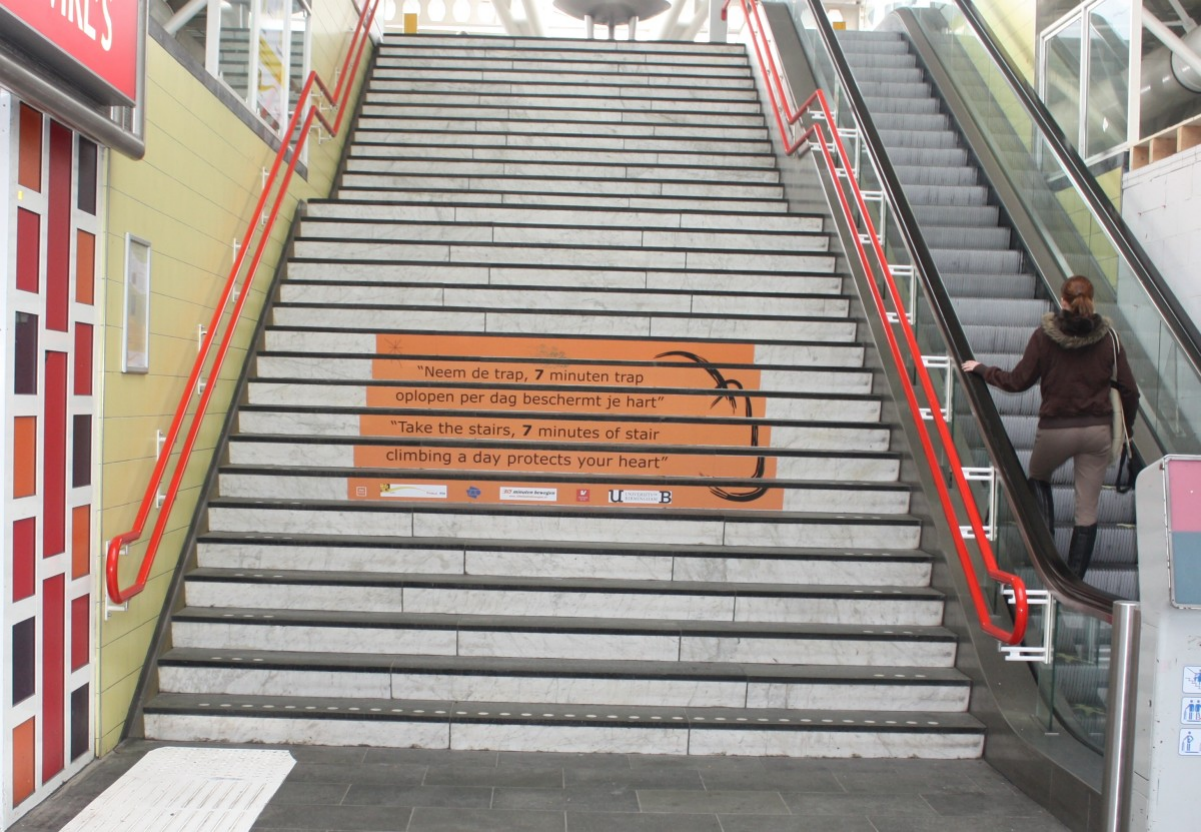
Intervention design based on Miro installed on the stairs in Leiden, the Netherlands.

Two weeks of baseline was followed by five weeks of the *design plus message* phase with the text ‘*Take the stairs*. *7 minutes of stair climbing a day protects your heart*’ and its translation into Dutch ‘*Neem de trap*. *7 minuten trap oplopen per dag beschermt je hart*’. As in Barcelona, this text was superimposed on the background of the adaptation of the Miro painting, Gota d´aigua damunt la neu rossa, 1968 (Drop of water on pink snow) prepared by Josep M^a^ Rius Graells (see S2 File for the full image). In addition, the background included logos for the University of Birmingham, University of Vic, TNO, ProRail and Pola Graphics on the bottom section. The intervention was composed of six, 0.17m high x 2.46m wide self-adhesive banners affixed to six stair risers beginning at the 7th step from the ground such that the 1.02m high x 2.46m wide intervention was centred 1.62m above the ground (see Fig 2).

Four weeks after removal of the intervention, the background alone was reintroduced in the same location as the original intervention for three weeks. A second baseline monitoring was begun two weeks before the *design alone* phase and, due to an oversight, monitoring continued for a further eight days after removal of the design.

## Results

Table 3 summarises the escalator use during different phases for study 2 in which *design alone* followed *design plus message* phases and Table 4 summarises the analyses of the *design plus message* and *design alone* phases in Leiden. As can be seen, escalator use decreased in response to both interventions relative to their preceding baselines and there were main effects of pedestrian traffic volume and direction of approach.

**Table 3:** Percentage escalator use (95% CI) in each phase for study 2.

Site	Location	Cue type	Cue area	First Baseline	*Design + Message*	Second Baseline	*Design alone*	Third Baseline
Leiden (2008–2009)	Station	Art	2.5m^2^	87.2%^a^ (86.3, 88.1)	83.0% (82.1, 83.9)	85.4% (84.4, 86.4)	83.4% (82.5, 84.3)	86.1% (85.1, 87.0)

^a^ The table contains raw percentages, uncorrected for effects of demographic grouping and pedestrian traffic volume.

**Table 4:** Summary of the effects of the interventions on escalator use in Leiden station.

Variable	*Design+Message* OR^a^ (95% CIs)	*Design Alone* OR (95% CIs)
Intervention < Baseline	0.69*** (0.63,0.76)	0.84*** (0.77,0.92)
Pedestrian Traffic (continuous)	0.998*** (0.997.0.998)	0.997** (0.996,0.997)
Right > Left	1.75*** (1.58,1.92)	1.51*** (1.38,1.66)

^a^ OR = odds ratio, CIs = confidence intervals; Intervention < Baseline means that escalator use was less frequent during the intervention than the baseline period as the outcome variable in modelling was escalator use

* = *p* < .05

** = *p* < .01

*** = *p* < .001.

During the baseline, 85.0% used the escalator when approaching from the left and 90.6% when approaching from the right. Overall, escalator use was less frequent when the stairs were reached before the escalator, 82.6% (95% CI = 82.1%, 83.2%) than when they could be reached after the escalator 88.2% (95% CI = 87.6%, 88.8%). There were no interactions between installed cues and direction of approach (both *p*>0.49).

Table 5 summarises comparisons between the phases for escalator usage in analyses that controlled for effects of direction of approach, pedestrian traffic and demographics.

**Table 5:** Summary of the comparisons between the phases for escalator use in Leiden station.

Comparison	OR^a^ (95% CIs)
*Design + message* vs. *Design alone*	0.97 (0.89, 1.05)
1^st^ baseline vs. 2^nd^ baseline	0.79 (0.72. 0.88) ***
*Design + message* vs. 2^nd^ baseline	1.14 (1.04, 1.25) **
2^nd^ baseline vs. 3^rd^ baseline	0.97 (0.88, 1.07)

^a^ OR = odds ratio, CIs = confidence intervals

* = *p* < .05

** = *p* < .01

*** = *p* < .001.

The first phase in each row is the reference phase in the analyses.

As can be seen, there was no significant difference in escalator use between the *design plus message* and *design alone* phases. During the second baseline, escalator use remained reduced relative to the initial baseline. Nonetheless, it was increased relative to the *design plus message* phase. Finally, there was no difference between the non-intervention periods either side of the *design alone* phase.

## Discussion

This study revealed that a *design alone* phase can disrupt habitual escalator choice after it has been paired with a health promotion message. Effects of health promotion had dissipated sufficiently to test for effects of the environmental cue alone. When the cue alone was removed, escalator use returned immediately to pre-cue levels. Consistent with the UK, commuters were more likely to choose the escalator if they reached it before the stairs [40].

For the shopping mall study presented in S2 File, reintroduction of the coloured background revealed no evidence for any effects of the *design alone*, despite obvious dissipation of disruptive effects of the original intervention. It was possible that the presence of the art background in the Leiden, rather than the difference between shopping and travel sites, accounted for the discrepancy. In the final study of the series, the Miro background was compared with a simple coloured background in two stations in Barcelona.

## Study 3

## Methods

The sites for study three (February—May, 2010) were two underground stations in Barcelona, Catalunya, namely Clot and Mundet. Both stations had adjacent stairs and an escalator (Clot, Art background, 39 steps, height of climb = 6.63m; Mundet, coloured background, 33 steps, height of climb = 5.28m) leading from the platform. Observations made from 8.00 am—10.00 am, two days a week (Clot *n* = 31,163; Mundet *n* = 40,433), were coded for each train to allow calculation of pedestrian traffic volume leaving the train for inclusion in the models.

### Interventions

**Fig 3:**
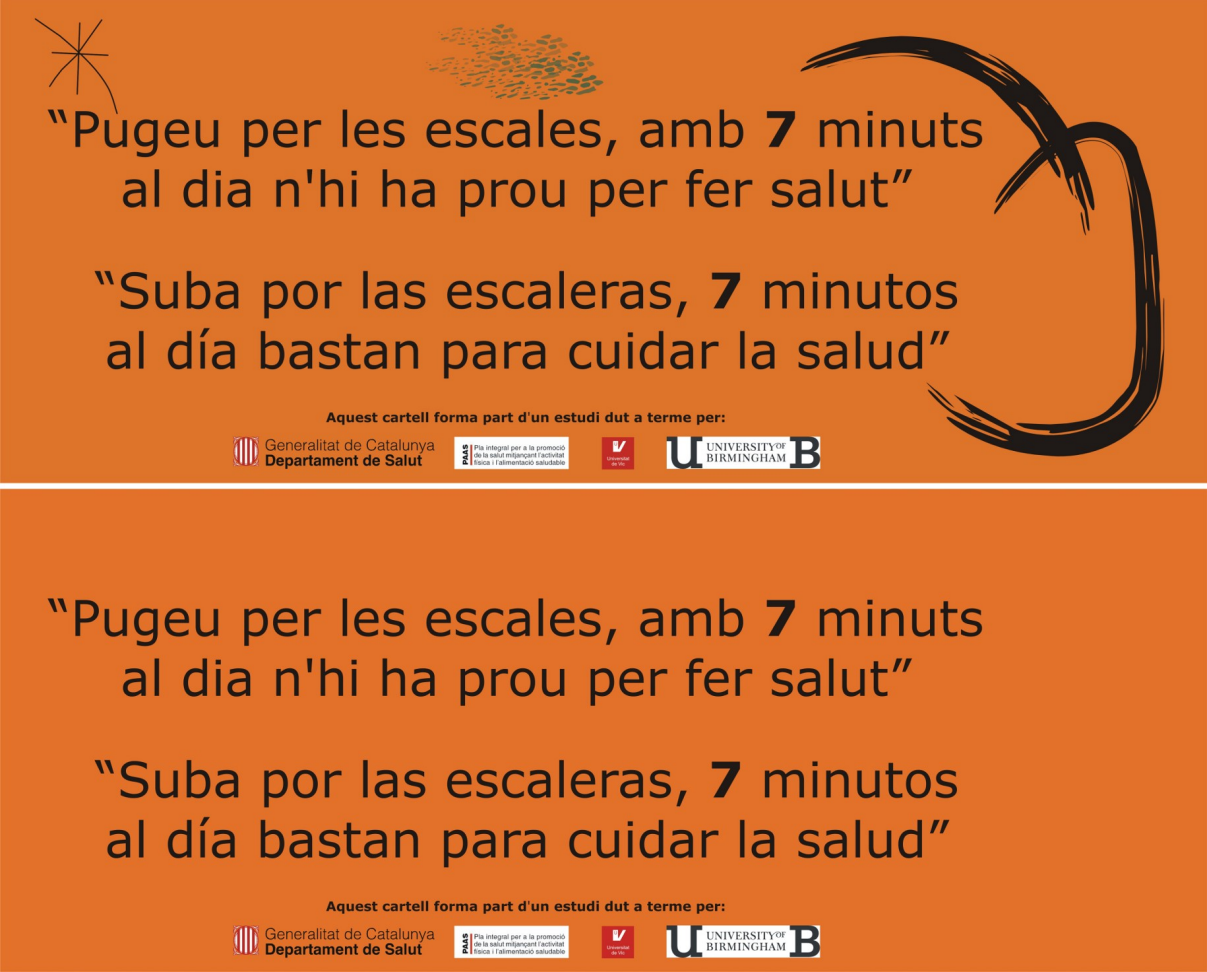
**A and B.** Intervention posters installed in metros stations in Barcelona; Clot (upper) and Mundet (lower).

A two-week baseline was followed by a six-week *design plus message* phase. The intervention was composed of six, 0.15m high x 2.25m wide self-adhesive banners affixed to six stair risers beginning at the 15^th^ step from the ground such that the 0.9m high x 2.25m wide intervention was centred 2.96m above the ground. The messages were translations into Catalan and Spanish of the English message ‘*Take the stairs*, *7 minutes of stair climbing a day protects your health*’ (translations Catalan; ‘*Pugeu per les escales*, *amb 7 minuts al dia n’hi ha prou per fre salut*’: Spanish; ‘*Suba por las escaleras*, *7 minuts al dia bastan para cuidar la salud*’). The Miro art background used previously was installed in Clot whereas Mundet used the orange background without the figurative elements of the Miro design. Both designs included logos for the University of Birmingham, University of Vic, the Department of Health, Catalunya, and PAAS (The Strategic Plan for Promoting Healthy Eating and Physical Activity) in Catalunya on the bottom section (see Fig 3).

Four weeks after removal of the intervention, the background without the messages was affixed to the same location on the stairs for the *design alone* phase for a further four weeks. The *design alone* phase was preceded by one week of baseline monitoring. As there were differences in average traffic volume between the stations (Clot mean = 38.9, *SD* = 13.5; Mundet mean = 73.1, *SD* = 36.2), traffic was mean centred for each station to avoid confounding intervention site with traffic in the omnibus analyses. The potential interactions between any installed signage and pedestrian traffic volume were tested.

## Results

**Fig 4:**
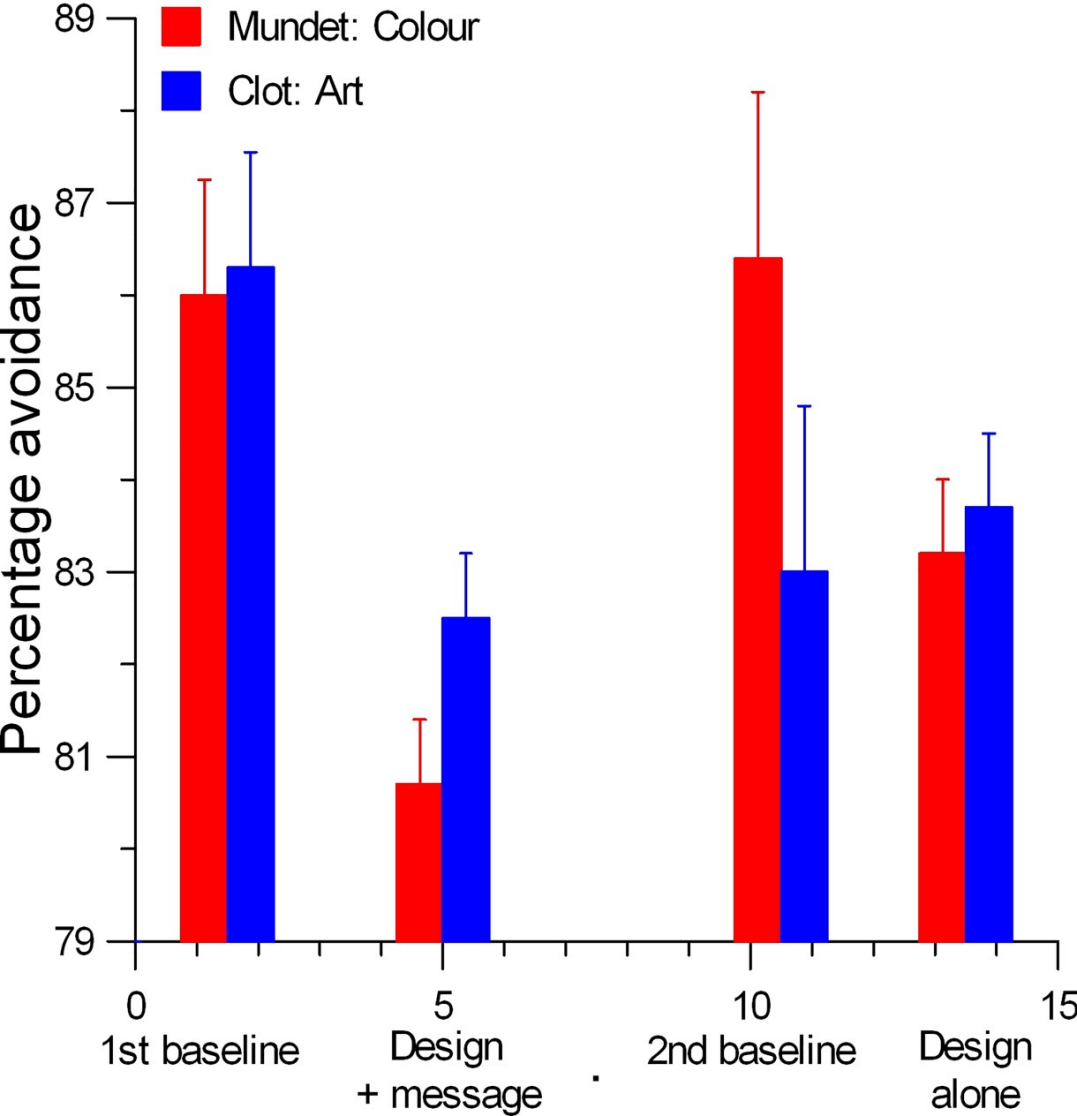
Summary of the percentage escalator usage during the different phase at Clot and Mundet stations in Barcelona.

During the baseline period, similar numbers used the escalator in the two stations [Clot; 86.3% (95% CI = 85.0, 87.4): Mundet; 86.0% (95% CI = 84.8, 87.0)]. Fig 4 summarises the percentage escalator usage (95% CIs) during the different phases at Clot and Mundet. Note that the bars are positioned at the week representing the centre of the intervention period and their width does not reflect the duration of that period.

Preliminary omnibus analyses of all the data revealed differential effects of station (*p* = .001) and its interaction with the intervention for the *design plus message phase* (*p* = .02), with similar effects during the *design alone* phase (*p* < .001 and *p* = .007 respectively). Therefore, separate analyses were computed for each station, summarised in Table 6.

**Table 6:** Summary of the effects of the interventions on escalator use in Clot and Mundet stations in Barcelona.

Variable	*Design + Message* OR ^a^ (95% CIs)		*Design Alone* OR (95% CIs)	
	Clot	Mundet	Clot	Mundet
Intervention < Baseline	0.74*** (0.67,0.82)	0.64*** (0.58,0.70)	1.07 (0.95,1.20)	0.82*(0.68,0.98)
Pedestrian traffic (continuous)	0.976*** (0.969,0.983)	0.981*** (0.979,0.984)	0.986** (0.976,0.996)	0.991*** (0.986,0.996)
Pedestrian traffic x Intervention	1.009* (1.001,1.017)	1.007*** (1.004,1.009)	1.012* (1.001,1.023)	0.995 (0.990,1.000)

^a^ OR = odds ratio, CIs = confidence intervals; Intervention < Baseline means that escalator use was less frequent during the intervention than the baseline period as the outcome variable in modelling was escalator use

* = *p* < .05

** = *p* < .01

*** = *p* < .001

As can be seen from Fig 4, escalator use was decreased during the *design plus message* phase in both stations with a numerically greater reduction at Mundet, the station with the coloured background, consistent with the intervention x station interaction in the omnibus analysis. For the *design alone* phase, however, reduced escalator usage at Mundet contrasted with no significant change at Clot. Table 7 summarizes the comparisons between the phases for escalator use at Clot and Mundet stations in analyses that controlled for the effects of the demographics and pedestrian traffic volume.

**Table 7:** Summary of the comparisons between the phases for escalator use at Clot and Mundet stations in Barcelona.

Comparison	Clot OR^a^ (95% CIs)	Mundet OR (95% CIs)
*Design + message* vs. *Design alone*	1.12 (1.05, 1.20) ***	1.17 (1.10, 1.23) ***
1^st^ baseline vs. 2^nd^ baseline	0.76 (0.67. 0.87) ***	1.04 (0.86, 1.23)
*Design + message* vs. 2^nd^ baseline	1.04 (0.93, 1.17)	1.50 (1.26, 1.79) ***

^a^ OR = odds ratio, CIs = confidence intervals

* = *p* < .05

** = *p* < .01

*** = *p* < .001.

The first phase in each row is the reference phase in the analyses.

Comparisons between the *design plus message* and *design alone* phases revealed reduced disruption of escalator use during *design alone* at both Clot and Mundet. During the second baseline, disruptive effects of the *design plus message* phase had dissipated at Mundet such that it was similar to the original baseline. In contrast, the second baseline at Clot had reduced rates of escalator use relative to the first. Consistent with this, escalator use at the second baseline were similar to the *design plus message* phase at Clot but increased relative to the intervention at Mundet.

In addition, escalator use was reduced at higher levels of pedestrian traffic and the significant interactions between pedestrian traffic and the interventions all reflected less disruption of choice by the intervention at higher traffic volumes (see S2 File for discussion of the effects of pedestrian traffic on interventions).

## Discussion

In the final study, re-introduction of the coloured background in Mundet disrupted escalator choice after a 6-week pairing with the health promotion message. The failure of colour to disrupt choice after pairing in the shopping mall (S2 File) may reflect insufficient pairings of the visual cue with the verbal message for that study’s population. For Clot, however, the disruptive effects of the health promotion message had not dissipated at the second baseline and, hence, the site was unable to test effects of the environmental cue alone. As only some pedestrians change behaviour with health promotion signage, maintenance of the effects of an intervention after its removal, as has been reported previously [24,53], may preclude further changes following the reintroduction of the background cue. Explicit knowledge acquired from the message during learning cannot be removed when the signage that provide it is taken down. In both stations, *design alone* disrupted escalator usage less than the *design plus message* phase, unlike in Leiden. One further difference between these Barcelona stations merits comment. Greater magnitude effects of the *design plus message* at Mundet may reflect the reduced height of the climb compared to Clot that pedestrians were encouraged to attempt. As noted earlier, avoidance of climbing is less likely, the lower the height of the climb [38,39].

## Overall discussion

Two initial studies revealed that large scale changes to the appearance of the environment did not change escalator use. When health promotion messages were added to the designs, escalator use was disrupted. In two subsequent studies, the *design alone* disrupted escalator use after it had been paired with a successful stair climbing campaign. There was no evidence that the art background was a more effective visual cue than simple coloured strips. This replicated series of studies in different countries clearly demonstrated that transport-related walking can be linked to environmental cues *that are encountered almost daily*. The following discussion focuses on a) the mechanisms underlying this learning and b) the environmental cues to which this learning could be linked.

### Learnt disruption of habitual choice

The sensitivity to cues means that commuter choices within the environment may be a fruitful paradigm to investigate habitual choice. Although learning of a cue-response pairing can be tested within a single laboratory session, life is better construed as a series of episodes, e.g. going to the bank then going for lunch. Each episode is separate, occurring in a different context from its predecessor. Thus, comparisons between episodes may better approximate the process whereby contextual cues are linked to real-world behaviours; entering the new context exposes a participant to the cues (c.f. [4,5,7]). For commuting pedestrians, each repeated episode is unique for that day, isolating its cues from more frequent daily repetitions. Single daily repetitions are the default design for habit learning with animals (e.g. [54]) and uniqueness of contextual cues within that day can only help experimental tests of their change. As the processing that underlies habits may be primarily in the instigation of behaviour by the context [55], the single daily exposure of commuters may be a good test of the model. Habits reflect automatic processes, directly cued by contexts, independent of any motivations that may have prompted the initial behaviour [7,32–34,56,57]. For rats, extensive repetitions, i.e. 500, produce this independence [57]. While the original response to the verbal message here reflects deliberative processing, sufficient pairings imbue the contextual cues themselves with effects of health promotion. Commuter studies with more pairings should be informative.

Despite extensive research in animals (see [58]), surprisingly, only three previous experimental demonstrations of environmental cue learning across days in humans are available. Conditioned tolerance to the effects of alcohol relative to placebo was learnt in three days when the environments differed in colour and furniture [59], echoing weaker effects resulting from repetitions over five days using different rooms in an earlier study [60]. Changes to recycling with an environmental cue in Holland et al (2006) were instantaneous [35]. The restricted number of pairings required for learning in the human studies above indicates an explicit basis for these effects. Explicit learning can be contrasted with implicit learning during which individuals are not aware that they are acquiring knowledge [61–63]. For laboratory studies, explicit learning of meaningful, real-world scenes required four repetitions whereas implicit learning for meaningless contexts required 10–15 repetitions [64,65]. An effect of learning after 3–5 days of pairing makes it likely to be explicit. Nonetheless, explicit and implicit learning occurs in parallel; implicit learning is an inevitable by-product of experience [62,63]. Participants in the human studies above would have acquired implicit knowledge that was incomplete at the time of testing.

The *design plus message* interventions specifically used deliberative, explicit processing to disrupt behaviour. Thirty pairings would produce explicit and implicit learning, with the latter achieved incidentally [64]. It could not, however, establish a new habitual response to the visual cue alone (see below). Rather, the environmental cue functions as a memory aid that re-elicits the deliberative processing that was originally engaged when the cue formed the background to the health promotion message. Deliberative processing could be primed by implicit processes and/or re-evoked explicitly by the contextual cue [27]. The no-intervention phase after *design alone* in Leiden is informative. Maintenance of disruption when a health promotion intervention is removed reflects explicit memory that decays gradually [24–26]. Following *design alone* in Leiden, however, disruption of escalator choice returned immediately to pre-cue levels. Such an immediate decay would be inconsistent with the more gradual decays seen in explicit memory. Some implicit priming by the cue seems likely; implicit priming by contexts has been reported previously [32]. Any daily user in Leiden would be exposed to 25 parings of the visual cues with the health promotion message. Extrapolating from the 77% of those interviewed during the commuting period who reported daily use (Eves, Lewis, Engbers & Puig-Ribera, in preparation) suggests an average of 19 pairings. This was sufficient for some implicit learning of the visual cue. (Eves et al., in preparation).

In the only previous cue-learning study of habitual physical activity, participants chose a novel health behaviour with a self-selected, daily cue [31]. Development of habitual processes in the exercise group (median = 91 days) ‘took one and a half times longer’ (page 1007 [31]) than development of eating or drinking habits. The greater complexity of the exercise behaviour may be relevant [31]. The habitual choice disrupted here was simpler, with the stair alternative making minor additional demands on time or effort compared to, for example, ‘running for 15 minutes before dinner’ (page 1000 [31]). In addition, commuters have many pairings in which to develop habitual choice. On average, individuals remain in the same job for over two years [66], with the median individual in the station studies (Spain, the Netherlands) having over eight months, i.e. 170 working days, of potential parings. Disruption of escalator choices of commuters may be a fairly stringent test for effects of health promotion on established habits.

### Environmental cues for transport-related walking

Although the studies here changed a single environmental cue, behavioural contexts encompass a much broader range. In Lally and co-workers’ elegant study, participants chose seventeen different cues (Lally personal communication, September 2014). The self-selected cues specified events, i.e. after breakfast, that might be tied to a specific environment but could also be location-independent, i.e. with lunch. Contextual cues have fuzzy boundaries [67]. Location, time, presence of others, current mood and prior action in a sequence have all been proposed as potential cues, with location the most prominent [7]. Nonetheless, the functionality of a location inextricably confounds its cues with an event that occurs within it. Where you work, and when you arrive there, can involve others and any mood that the event engenders. Contexts are episodes of life in which there can be the simultaneous operation of multiple cues, only some of which concern the physical location itself. The studies here changed a single visual cue *within* a repeated travel episode, demonstrating effects of a cue independent of this contextual soup.

There are three other instances of effects of contextual cues on transport-related walking. Climate influenced choice to walk up a moving travellator in Hong Kong; increased humidity which would impair physiological resources reduced walking [68,69]. While the potential cue of time of day influences choice [40,44,68,69], it may be inextricably confounded with pressure to reach work on time [40,44] and travelling during working hours [68,69]. In addition, behaviour of others can disrupt escalator use. Seeing a preceding pedestrian choosing to climb stairs influenced pedestrians following them down a 51m corridor on the approach to the stair/escalator complex in a shopping mall [70]. An overall +1.3% increase in stair climbing when a natural role model was visible during the approach is of considerably lower magnitude than explicit effects of health promotion messages at this site (sample size weighted average = +8.1%; *n* = 50,609 [24,47]). Where the cued behaviour entailed costs, i.e. climbing, effects were considerably lower that when it provided the benefit of reducing journey time by jaywalking (+19.6%; see [71]). No information on prior actions or mood is available.

### Limitations

We have reasoned that choice of the escalator becomes habitual but without interviews, the actual frequency of choice, and hence its habitual nature, is unknown. Nonetheless, high baseline rates of escalator use (99.3%-86.0%) appear inconsistent with a population that alternates randomly between the escalators and stairs at an individual level. Further, pedestrian behaviour is a noisy quasi-experimental paradigm. Demographic composition of the sample at any time point is uncontrolled (see S1 File), as is the flow of pedestrian traffic (see [44], supplemental materials). Demographic coding is imperfect, despite excellent Kappas for the test, and inclusion of these factors in analyses only provides partial control. Nonetheless, choice paradigms audit actual behaviour with minimal demand characteristics on the pedestrians or any self-presentation concerns arising from self-report. This real behaviour is accurately measured. This series of studies employed seven different sites and three different behavioural contexts, namely outdoor transport, shopping and travelling to work. The minor differences in stair height between Mundet and Clot were reflected in minor differences in intervention efficacy as could be expected from the effects of height of the potential climb on choice [38,39]. Despite this, the health promotion messages disrupted escalator use in *all* sites suggesting a commonality between the pedestrians. Further, the weighted average effects of disruption by point-of-choice prompts in stations, +5.2% (*n* = 336,681; Eves, unpublished), appears comparable to the disruptive effects here (c.f. Table 3 and Fig 4). Importantly, the studies were not about the messages or the sites themselves but rather whether learning of contextual cues could occur when transport-related walking was the behaviour. Although the sites may differ, the underlying behaviour was the same; all studies disrupted pedestrian choice and cues alone could disrupt choice after pairing.

### Conclusions

In summary, these studies demonstrated that changes to the environmental cues alone at a stair and escalator choice-point did not alter behaviour. If, however, the visual cue was paired with successful health promotion messages, then subsequent reintroduction of the cue alone could disrupt choice. To our knowledge, this represents the first experimental demonstration of learning of an environmental cue with a subsequent effect on habitual behaviour in humans. Hence, the widely held premise that successful behavioural choices can be linked to contextual cues was confirmed experimentally.

## Supporting information

S1 File Effects of demographic grouping on escalator use. (PDF)

S2 File Preliminary tests of the effects of visual cues on transport-related walking. (PDF)
