# Supplementary material for: Learnt effects of environmental cues on transport-related walking; disrupting habits with health promotion?
Source: PLoS One. 2019 Aug 1;14(8):e0220308. doi: 10.1371/journal.pone.0220308 (PMC6675111; doi:10.1371/journal.pone.0220308)
Supplement: S1 File — (PDF) [file pone.0220308.s001.pdf]

Supporting File 1

Effects of Demographic grouping on escalator use

Choice between stairs and escalators varies by demographic grouping. Typically, women, those appearing over 60 years old, non-white individuals, those carrying large bags or a push chair and those accompanied by children avoid the stairs by choosing the escalator more than their comparison groups [Eves, 2014; Kerr, Eves & Carroll, 2001; Webb, Eves & Kerr, 2011]. Additionally stair avoidance is less frequent at higher levels of pedestrian traffic volume [Kerr et al., 2001; Webb et al., 2011]. Neither demographic composition of the sample nor pedestrian traffic volume can be experimentally controlled; the 'participants' are pedestrians negotiating the built environment at that point in time. Hence, for all studies, Odds Ratios (OR) from multiple logistic regression must be the basic unit of evidence as these represent the effects of the cues corrected for the uncontrollable influences of demographic composition of the sample and pedestrian traffic volume. Information about the coded demographics for each study, and hence the demographics included in any analyses, is provided below.

Demographic composition of the sample in each study

Study 1

For the coded observations (N = 5,987), 50.3% were women, 85.9% were classified as under 60 years old and 8.0% carried large bags.

In analyses, there were no significant effects of the demographic variables for this relatively small sample.

Study 2

For the coded observations (N = 37,479), 53.2% were female and 61.0% approached from the left and, hence, reached the stairs before the escalator. Double coding with a second observer revealed average Kappas of 0.85 (range = 0.81 - 0.90). As expected from the more even spacing of pedestrians over time, increased traffic was not associated with reduced effects of the intervention and the interactions were dropped from the final models.

In the analyses, females used the escalator more than men during the design plus message phase (OR = 1.20, 95% CI = 1.10, 1.31, p<.001) and during the design alone phase (OR = 1.25, 95% CI = 1.15, 1.37, p<.001).

Study 3

For the coded observations (Clot n = 31,163; Mundet n = 40,433), 63.8% were female, 96.7% were classified as under 60 years old, 88.6% classified as white and 0.8% carrying large bags (average Kappa = 0.84, range = 0.82 - 0.92).

Concerning these uncontrollable influences on choice, generally, women, older pedestrians, non-white individuals and those with large bags used the escalator more than their comparison groups consistent with previous research (see table 1 below).

Table 1: Summary of the effects of the demographics on escalator use in Clot and Mundet stations during each phase.

Variable	*Design + Message* OR[a] (95% CIs)		*Design Alone* OR (95% CIs)	
	Clot	Mundet	Clot	Mundet
Females > Males	1.06	1.45***	1.40***	1.66***
	(0.98, 1.15)	(1.34, 1.57)	(1.27, 1.54)	(1.51, 1.82)
Non-white >	2.64***	1.96***	3.62***	2.73***
White	(2.34, 2.99)	(1.56, 2.45)	(3.06, 4.27)	(2.04, 3.66)
Old > Young	3.76***	2.14***	5.32***	2.93***
	(2.78, 5.07)	(1.59, 2.88)	(3.41, 8.29)	(2.09, 4.10)
Bags > No bags	2.06**	3.56***	7.20***	1.09
	(1.30, 3.28)	(1.66, 7.66)	(2.27, 22.8)	(0.45, 2.60)

[a] OR = odds ratio, CIs = confidence intervals; Females > Males means that escalator use was more frequent in women than men as the outcome variable in modelling was escalator use; * = $p<.05$, ** = $p<.01$, *** = $p<.001$

References

Eves FF: **Is there any Proffitt in stair climbing? A headcount of studies testing for demographic differences in choice of stairs.** *Psychonom Bull Rev* 2014, **21**:71-79.

Kerr J, Eves F, Carroll, D: **Six-month observational study of prompted stair climbing.** *Prev Med* 2001*,* **33**:422-427.

Webb OJ, Eves FF, Kerr J: **A statistical summary of mall-based stair-climbing interventions.** *J Phys Act Health* 2011*,* **8**:558-65.
