# Supplementary material for: Learnt effects of environmental cues on transport-related walking; disrupting habits with health promotion?
Source: PLoS One. 2019 Aug 1;14(8):e0220308. doi: 10.1371/journal.pone.0220308 (PMC6675111; doi:10.1371/journal.pone.0220308)
Supplement: S2 File — (PDF) [file pone.0220308.s002.pdf]

Supporting File 2

Preliminary tests of the effects of visual cues on transport-related walking

A companion study, contemporaneous with the study in the outdoor site in Barcelona, was conducted in a shopping mall in Coventry, England (September – October, 2004). As details of the study have been reported elsewhere [Webb & Eves, 2007a], here we summarise the methods to clarify the art-based design and the results for the *design alone* phase on ascending pedestrians on the intervention staircase, and then provide details of previously un-reported analyses comparing the different phases.

Study 1

In this shopping mall, a central bank of escalators was flanked by two 15-step staircases (height of climb = 2.55m).

Methods

Following a three-week baseline phase, a colourful design using red, white, yellow, blue and black strips was installed so that it completely covered the stair risers on the staircase adjacent to the up escalator. The total design was 2.55m high x 1.4m wide and in the style of Mondrian (see figure 1). Three weeks later, the messages '*Take the stairs*' and '*7 minutes of stair climbing daily protects your heart*' were superimposed twice over the design [see figure 1 in Webb & Eves (2007a) for the positions of the text on the image]. Observations were made twice weekly, between 12.30pm and 4.00pm with a pool of trained observers (N = 40,057). For the coded observations 56.2% were female, 75.4% were classified as White, 93.3% were classified as under 60 years old and 7.3% carried large bags (inter-observer agreement = 97%, range = 100% - 94%).

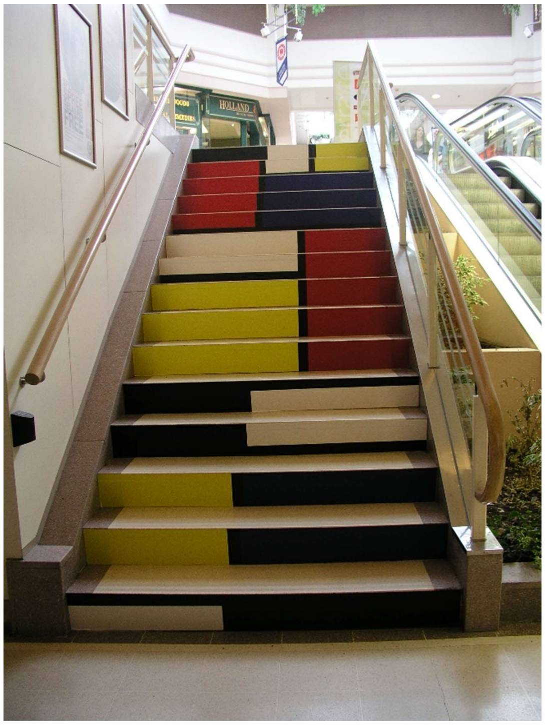

Figure 1: Intervention design based on Mondrian used in the station study in Coventry.

Results

During the baseline period, 96.3% chose the escalator. Bootstrapped analysis confirms there was no disruption of escalator choice by the *design alone* phase reported previously (OR = 1.13, CIs = 0.99, 1.29, p=.08). In the new analyses, addition of text to the design decreased escalator use relative to the baseline (OR = 0.43, CIs = 0.38, 0.48, p<.001) and escalator use during the *design plus message* phase was less than during the *design alone* phase (OR = 0.37, CIs = 0.33, 0.42, p<.001). As is clear from this study, and the preceding one in the main paper, there was no evidence for effects of *design alone* on escalator choice.

Below we summarise the design and results from two initial attempts to test the potential effects of visual environmental cues, i.e. *design alone*, on escalator use after they have been paired with a verbal health promotion message. The effects of the verbal health promotion phase, *design plus message*, have been reported previously for the shopping mall study

(Webb & Eves, 2007) and for the initial attempt to test *design alone* in a train station in the Barcelona underground (Puig-Ribera & Eves, 2010). In contrast, the effects of *design alone* are reported here for the first time. In addition, we discuss at length the difference between shopping mall and train station pedestrian traffic flows and the implications these can have for interventions.

Additional study 2

Study two was conducted in a shopping mall in Birmingham, England (November, 2004 - March, 2005) where a 13-week *design plus message* phase was followed by a one-week *design alone* phase beginning five weeks after the main intervention. Although the main results of this study have been reported elsewhere (Webb & Eves, 2007b), we report here for the first time effects of the *design alone* phase after pairing with verbal health promotion and information about the visual cues that formed the *design alone* phase.

Methods

In summary, the study site contained a 24-step staircase (height of climb = 4.08m) flanked by adjacent escalators. Inconspicuous observers recorded whether individuals chose the stairs or escalator, coding their gender, age, skin colour and presence of large bags using the standard protocol from previous research (Kerr, Eves & Carroll, 2001; inter-observer agreement = 97%, range = 100%-94%). Observations were made between 12.30pm and 4.00pm on two days of any week in which monitoring took place.

A two-week baseline was followed by 13 weeks in which message banners were installed on the stair risers. The message '*Take the stairs*' was presented twice superimposed on a white background. Additionally, the messages, '*Stair climbing burns more calories per minute than jogging*', '*7 minutes of stair climbing per day protects your heart*' and '*Stair climbing burns more calories per minute than tennis*' were presented on blue, red and blue backgrounds respectively, with each message requiring three stair risers. In total, the design

covered 11 stair risers resulting in a total design that covered 1.87m in height and 1.3m in width (see figure 1, Webb & Eves, 2007b). Five weeks after removal of the main intervention, the coloured backgrounds of the messages were reintroduced on the stair risers. One week of observations preceded this *design alone* phase. The analyses reported here employed logistic regression with bootstrapping with replacement to control for potential non-independence of the observations (samples = 1000). Escalator/stair use was the dichotomous outcome variable and intervention phase, gender, age, skin colour, presence of bags and pedestrian traffic volume the potential predictor variables.

Results

For the coded observations (*N* = 32,583), 54.4% were female, 68.3% were classified as White, 84.0% were classified as under 60 years old and 9.9% carried large bags. During the baseline period, 94.7% chose the escalator in this shopping mall. In the last week of the intervention (week 13), escalator use was reduced relative to baseline (OR = 0.40, CIs = 0.35, 0.47, $p<.001$). In the second baseline period preceding the *design alone* phase, some reduction in escalator use remained relative to the original baseline (OR = 0.60, CIs = 0.49, 0.72, $p<.001$). Nonetheless, escalator use was increased relative to the intervention phase (OR = 1.49, CIs = 1.25, 1.76, $p<.001$). Critically, introduction of the *design alone* had no effect on escalator use (OR = 1.01, CIs = 0.83, 1.22, $p=.92$). Additionally, these analyses consistently contained significant main effects of gender, age, skin colour and pedestrian traffic volume (all $p<.01$).

Discussion

Additional study two revealed no effects of contextual cues after pairing with health promotion messages, despite increased rates of escalator use relative to the intervention at the second baseline. Choice of a shopping mall for the intervention site may have been sub-optimal. The number of repetitions required to link contextual cues to behaviour is so far unknown. For pedestrians in a shopping mall, it is unclear how many times they would use

the site and insufficient pairings of the contextual cues with the message may have occurred, despite the 13-week intervention. Thus, the next study was conducted in a train station, with coding of pedestrian choices in the morning during the commuting period.

For station studies, one point should be made explicit. In train stations, baseline rates of escalator use are typically lower than in shopping malls (Eves, Lewis, & Griffin, 2008; Eves, Olander, Nicoll, Puig-Ribera, & Griffin, 2009; Eves & Webb, 2006). An updated running total from 17 studies (combined n = 336,681), estimates average baseline escalator use, weighted by sample size, at 83.7% (95% CI = 83.6, 83.8), considerably less frequent than the average in shopping malls, 92.4% (95% CI = 92.3, 92.5; 15 studies, combined n = 355,069; Eves, unpublished). The pulsatile nature of pedestrian traffic flow in train stations means that many disembarking passengers seek to leave the station at the same time. If the escalator is blocked by preceding pedestrians, the stairs represent a quicker available route out of the station. Consequently, passengers will choose the stairs, reducing rates of escalator use compared to shopping malls with traffic flows more evenly distributed over time (Olander & Eves, 2008; Lewis & Eves, 2012). There is an important corollary of variations in pedestrian flow in stations; effects of interventions appear reduced at higher traffic volumes for relatively complex messages (Olander & Eves, 2008; Lewis & Eves, 2012). In particular, Lewis & Eves (2012) reported reduced effects for the ‘more complex’ message, *Regular stair climbing for 7 minutes per day protects your heart*, a message with similar complexity to those used here. Further, higher rates of stair climbing due to pulsatile flow can mean that an intervention installed on the stair risers could sometimes be partially obscured by the legs of pedestrians leaving the station ahead of the perceiver. As a result, the analyses for the station studies tested for interactions between the signage and pedestrian traffic volume.

Additional Study 3

Although a brief summary of the intervention in this study has been reported previously (Puig-Ribera & Eves, 2010), this paper reports for the first time effects of the *design alone* phase after pairing and formally compares it with first and second baseline periods and the

intervention that immediately preceded it. Observations covered the main commuting period in Barcelona, i.e. 8.00 – 10.00 am, to maximize the number of pairings and, hence, potential learning of cues. Effects of pedestrian traffic volume and its potential interaction with any intervention were included in the modelling.

Methods

The site for additional study three was an underground station, Passeig de Garcia, in Barcelona, Catalunya in Spain (February - May, 2006). Two separate platforms discharged passengers into a concourse where an adjacent escalator and 24-step staircase led out of the station (height of climb = 3.84m). Additionally, the stairs from a lower level metro line in the station discharged passengers next to the escalator.

Two inconspicuous observers (average Kappa = 0.88, range = 0.82 - 0.92) coded stair and escalator choices of ascending pedestrians as well as their gender, age, skin colour, presence of large bags and presence of accompanying children using the standard protocol (Kerr et al, 2001a). Observations made from 8.00 am - 10.00 am, two days a week were coded into separate 30 minute periods to allow calculation of pedestrian traffic volume for inclusion in the models.

Interventions

A two-week baseline was followed by a six-week *design plus message* phase. The intervention was composed of six, 0.16m high x 2m wide self-adhesive banners affixed to six stair risers beginning at the 15th step from the ground such that the centre of the design was 2.72m above the ground. The message phase analysed here were the translations into Catalan and Spanish of the English message '*Take the stairs! 7 minutes of stair climbing a day protects your health*' (translations Catalan; '*Pugeu per les escales, amb 7 minuts al dia n'hi ha prou per fre salut*': Spanish; '*Suba por las escaleras, 7 minuts al dia bastan para cuidar la salud*').

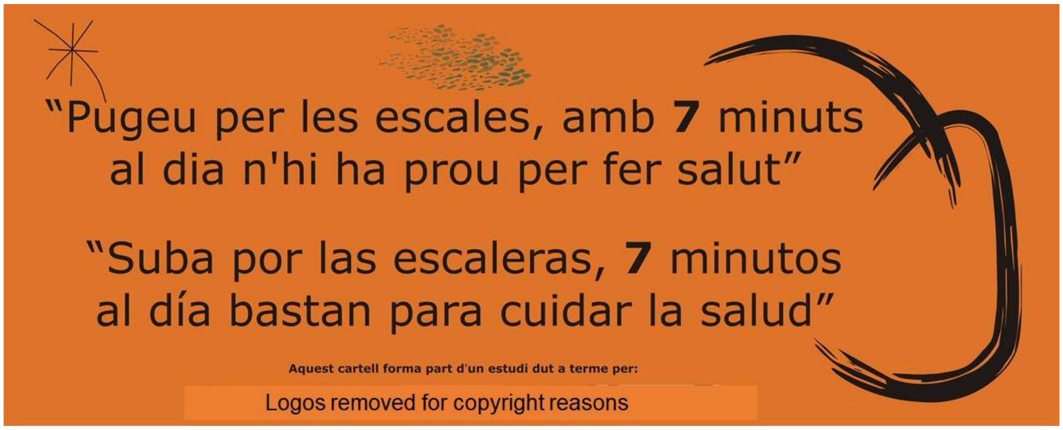

Figure 2: Intervention design based on Miro used in the station studies (with message used in Barcelona).

Figure 2 depicts the design used for this study and subsequent studies, with figure 3 showing its installation in the station. The *design alone* was based on a relatively minimal Miro painting (Gota d´aigua damunt la neu rossa, 1968: Drop of water on pink snow) such that an orange background contained the two large, black free hand abstract shapes positioned to the right of the message. This adaptation was prepared by Josep Mª Rius Graells.

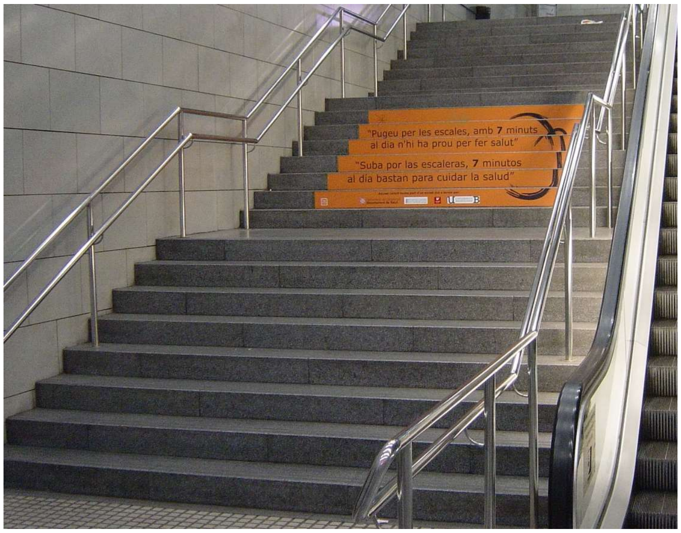

Figure 3: Intervention design based on Miro installed on the stairs in Passeig de Gracia metro station, Barcelona.

The whole intervention measured 0.99m high x 2m wide. In addition, the background included logos for the University of Birmingham, University of Vic, the Department of Health, Catalunya, PAAS (The Strategic Plan for Promoting Healthy Eating and Physical Activity) in Catalunya and Pola Graphics on the bottom section.

Three weeks after removal of the intervention, the background without the messages was affixed to the same location on the stairs for the *design alone* phase and observations made for two weeks. The *design alone* phase was preceded by one week of baseline monitoring. As the aim was to assess effects in commuters, children and individuals accompanied by children were excluded from analyses. The analyses reported here employed logistic regression with bootstrapping with replacement to control for potential non-independence of the observations (samples = 1000). Escalator/stair use was the dichotomous outcome variable and intervention phase, gender, age, skin colour, presence of bags and pedestrian traffic volume the predictor variables. In addition, the potential interaction between any installed signage and pedestrian traffic volume was tested.

Results

For the coded observations (N = 32,276), 63.8% were female, 93.6% were classified as under 60 years old, 97.1% as white and 1.0% carrying large bags. During the baseline period, 94.2% used the escalator (see discussion below). The *design plus message* phase with the 7 minutes of stair climbing a day message reduced escalator use (OR = 0.66, CIs = 0.56, 0.78, p<.001), with less reduction by the intervention at higher pedestrian traffic volumes (OR = 1.003, CIs = 1.001, 1.005, p=.002). In the second baseline period preceding the *design alone* phase, escalator use remained reduced relative to the original baseline (OR = 0.81, CIs = 0.67, 0.98, p=.03) but was increased relative to the intervention phase (OR = 1.22, CIs = 1.01, 1.48, p=.05). Critically, the *design alone* phase was associated with reduced escalator use relative to the original baseline (OR = 0.75, CIs = 0.64, 0.89, p<.001) but not relative to the second baseline immediately preceding it (OR = 0.96, CIs = 0.80, 1.15, p=.66). Nonetheless, the *design alone* phase was not significantly different from the *design*

plus message phase (OR = 1.09, CIs = 0.95, 1.25, *p*=.25). These analyses of the *design alone* phase did not contain significant interactions between traffic volume and effects of the intervention (all *p*>.15). As reported previously, generally, the analyses contained significant main effects of gender, age, skin colour and pedestrian traffic volume (Puig-Ribera & Eves, 2010).

Discussion

This study provided only weak evidence that a visual contextual cue could disrupt escalator choice. While disruption during the *design alone* phase did not differ statistically from that during the *design plus message* phase, it was no more frequent than during the second baseline phase. In this study, the second baseline period was begun only two weeks after removal of the intervention and disruptive effects of the *design plus message* may not have dissipated when the *design alone* phase was introduced. As noted in the introduction to this section on the effects of *design alone* after pairing with messages, if rates of disruption remain elevated relative to the original baseline, testing for effects of cues alone is more difficult. In the next study in the main paper, the gap between the *design plus message* and *design alone* phases was increased to four weeks to reduce carryover effects of disruption by the verbal health promotion message.

One further point about pedestrian behaviour from this study is important. Layout of the environment will bias choice independent of any intervention. Three out of the four platforms at Passeig de Gracia discharged passengers next to the escalator. An escalator reached first is more likely to be chosen (Eves et al., 2009). For two of these platforms, a prior climb to reach the escalator would further discourage choice of the stairs. The net outcome in Passeig de Gracia was that baseline percentages of escalator use were elevated (94.2%, 95% CI = 93.5, 94.9) such that they overlapped with those in the shopping mall in the UK reported in Additional study 1 (94.7%, 95% CI = 94.1, 95.4), despite appreciably lower rates in stations than shopping malls in previous research (83.7% vs. 92.4%; combined *n* = 691,750, Eves, unpublished). Inevitably, this bias towards escalator use resulting from the

layout of Passeig de Gracia station reduced absolute effects of the health promotion intervention (see Puig-Ribera & Eves, 2010) and would militate against testing for effects of the *design alone* phase.

For stations in the Barcelona underground, the stairs and escalator represent the only exit from the platform, like stations investigated in the UK (e.g. Eves et al., 2009). In the next study, reported in the main body of the paper, we used a station in Leiden, the Netherlands. At Leiden station, the stairs and escalator lead up to the platform rather than away from it and potential passengers arrive at different times for the same train. As a result, pedestrian traffic is distributed more evenly over time. An arriving pedestrian is less likely to find the escalator blocked and preceding pedestrians are much less likely to impede visibility of any signage installed on the stairs. This more even flow of pedestrian traffic should reduce potential interactions between pedestrian traffic volume and the intervention noted above.

References

Eves FF, Lewis AL, Griffin, C: **Modeling effects of stair width on rates of stair climbing in a train station.** *Prev Med* 2008, **47**:270-272.

Eves FF, Olander EK, Nicoll G, Puig Ribera A, Griffin C: **Increasing stair climbing in a train station; effects of contextual variables and visibility.** *J Envir Psychol* 2009, **29**:300-303.

Eves FF, Webb OJ: **Worksite interventions to increase stair climbing; Reasons for caution.** *Prev Med* 2006, 43:4-7.

Kerr J, Eves F, Carroll, D: **Six-month observational study of prompted stair climbing.** *Prev Med* 2001, **33**:422-427.

Lewis A, Eves FF: **Prompts to increase stair climbing in stations; the effect of message complexity.** *J Phys Act Health* 2012, **9**:954-961.

Olander EK, Eves FF, Puig-Ribera A: **Promoting stair climbing: stair-riser banners are better than posters... sometimes.** *Prev Med* 2008, **46**:308-310.

Puig-Ribera A, Eves FF: **Promoting stair climbing in Barcelona: similarities and differences with interventions in English-speaking populations.** *Eur J Pub Health* 2010*,* **20**: 100-102.

Webb OJ, Eves FF: **Effects of environmental changes in a stair climbing intervention: Generalization to stair descent.** *Am J Health Prom* 2007a*,* **22**:38-44.

Webb OJ, Eves FF: **Promoting stair climbing: Intervention effects generalize to a subsequent stair ascent.** *Am J Health Prom* 2007b, **22**:114-119.
